# Pien Tze Huang regulates phosphorylation of metabolic enzymes in mice of hepatocellular carcinoma

**DOI:** 10.1038/s41598-023-29116-8

**Published:** 2023-02-02

**Authors:** Jinxia Lin, Shicong Wang, Wenliang Lan, Ming Ji, Mei Li

**Affiliations:** 1 Zhangzhou Pientzehuang Pharmaceutical Co., Ltd., Huporoad, Zhangzhou, 363000 People’s Republic of China; 2 Fujian Pien Tze Huang Enterprise Key Laboratory of Natural Medicine Research and Development, Zhangzhou, 363000 Fujian People’s Republic of China; 3 Institute of Materia Medica, Chinese Academy of Medical Sciences and Peking Union Medical College, Beijing, 100050 People’s Republic of China; 4 Department of Thoracic Surgery, Nanfang Hospital, Southern Medical University, Guangzhou, 510515 Guangdong People’s Republic of China

**Keywords:** Cancer metabolism, Pharmacology, Drug development

## Abstract

The Chinese medicine formula Pien Tze Huang (PZH) has been applied to the treatment of various diseases, the reported anti-tumor mechanisms included regulation of inflammation-associated cytokine secretion and cancer growth pathways. However, the potential influence of PZH on tumor metabolism remains unclear. This study aimed to investigate the global effect of PZH on hepatocellular carcinoma (HCC) compared with the anti-tumor agent sorafenib based on tandem mass tag (TMT) label proteomic and phosphoproteomic analysis in addition to parallel reaction monitoring (PRM) verification. It was observed that PZH could inhibit tumor weight by 59–69% in different concentrations. TMT proteomic studies indicated that fructose/mannose metabolism and glucagon signaling pathway in PZH group, and arachidonic acid metabolism and PPAR signaling pathway in sorafenib group, were significantly enriched, while glycolysis/gluconeogenesis pathway was found to be enriched remarkably both in PZH and sorafenib groups in TMT phosphoproteomic study. PRM verification further indicated that both PZH and sorafenib could down-regulate phosphorylations of the glycolytic enzymes phosphofructokinases 1, fructose-bisphosphate Aldolase A, phosphoglycerate mutase 2 and lactate dehydrogenase A chain, while phosphorylations of long chain fatty acid CoA ligase in fatty acid activation and acetyl-coenzyme A synthetase in glycolysis were significantly inhibited by PZH and sorafenib, respectively. This study proposed that PZH shared a similar anti-tumor mechanism of metabolic regulation to sorafenib, but differed in the regulation of some metabolic nodes. This is the first time to uncover the relationship between the anti-tumor effect of PZH and metabolic related enzymes, which distinguished from the known mechanisms of PZH. These data provided the potential molecular basis for PZH acting as a therapeutic drug for HCC, and offered cues of manipulation on Warburg effect under the treatment of PZH.

## Introduction

Liver cancer, the third leading cause of cancer death globally, is still a high-risk malignant tumor in China^1^. Primary liver cancer includes 75–85% cases of hepatocellular carcinoma (HCC) induced by risk factors hepatitis virus, alcohol, and obesity etc. Nowadays, multiple tyrosine kinase inhibitors such as sorafenib, and immune checkpoint inhibitors including nivolumab have been used to treat HCC. In addition, traditional Chinese medicine (TCM) has been proved as a beneficial choices for HCC patients^2–4^.

Metabolic reprogramming has been considered as a hallmark of cancer, but it remains poorly defined in HCC^5,6^. Warburg effect which illustrates that tumor cells tend to choose aerobic glycolysis over oxidative phosphorylation, is a well known metabolic change and plays a critical role in tumorigenesis and tumor progression. Currently, increasing attention on glycolytic enzymes as potential therapeutic targets was proposed due to their associations with cancer specific metabolism. Multiple glycolytic enzymes, such as PFK1 (phosphofructokinases) and ALDOA (fructose-bisphosphate aldolase A)^7,8^, have been reported to be overexpressed in HCC, and some studies demonstrated that the active aerobic glycolysis phenotype was associated with poor prognosis of HCC^9^, meanwhile, increases of glycolytic enzymes (aldolase, enolase, and pyruvate kinase) correlated with sorafenib resistance have been previously reported in HCC patients and cells^10,11^. Therefore, the above-mentioned proteins provide insights into promising metabolism-related therapeutic targets of HCC, and it was proposed that anti-Warburg therapies targeting key transporter and enzymes involved in glycolysis or their combination with multiple tyrosine kinase inhibitors will set the trend of advanced HCC therapy in the future^12,13^.

TCM has been practiced as a complementary and an alternative treatment for diverse cancers in China, with the effects of alleviating the tumor symptoms, improving quality of life, controlling tumor size and prolonging patient survival, as well as regulating immunity^4,14^. Traditional Chinese patent medicine Pien Tze Huang (PZH) mainly containing *Panax notoginseng*, *Moschus*, *Calculus Bovis and Snake Gall*^15^, was originated from the Chinese Ming Dynasty and had been officially approved to treat acute and chronic viral hepatitis and various inflammatory diseases in China. Recently, it was approved for drug clinical trials of advanced primary liver cancer by National Medical Products Administration of China (Approved number 2020LB00056). PZH has been used worldwide, especially in Southeast and Northeast Asia. Nowadays, more and more research has revealed that PZH could be applied to treat cancers based on clinical trials^16^, particularly in HCC to reduce tumor size and side effect, improve clinical symptoms and life quality, alleviate immunity inhibition^17–19^ and prolong overall survival (unpublished data). Meanwhile, its anti-HCC mechanisms were explained as anti-proliferative and anti-apoptotic effects in vitro^20^, and regulating inflammation-associated cytokine secretion and cancer growth pathways in vivo^21^. However, the metabolism-related information regarding to PZH treatement remains unknown while these have been documented in other TCM therapies^22,23^.

Mass spectrometry (MS) based technologies of tandem mass tag (TMT) quantitative proteomics and phosphoproteomics (hereafter collectively referred to as (phospho)proteomics) facilitate the global assessment of proteins and phosphorylation sites. Parallel reaction monitoring (PRM) considered as an antibody-free and MS-based western blot is usually applied for (phospho)proteomics data verification to reduce protein false positives of omics studies due to its advantages of higher selectivity and sensitivity, good reproducibility, and strong anti-interference ability^24^.

In this study, TMT (phospho)proteomics were performed to globally assess the biological consequences of PZH treatment compared to the widely accepted anti-HCC agent sorafenib on a mouse model, basing on the analysis of the relevant networks of PZH and sorafenib effects. PRM verification was further used to confirm the key nodes of drugs’ treatment. This work may bring more insight and understanding into the potential mechanisms from protein level and phosphorylation perspective involved in PZH treatment of HCC.

## Results

### Inhibition of xenograft tumor growth in vivo

**Figure 1:**
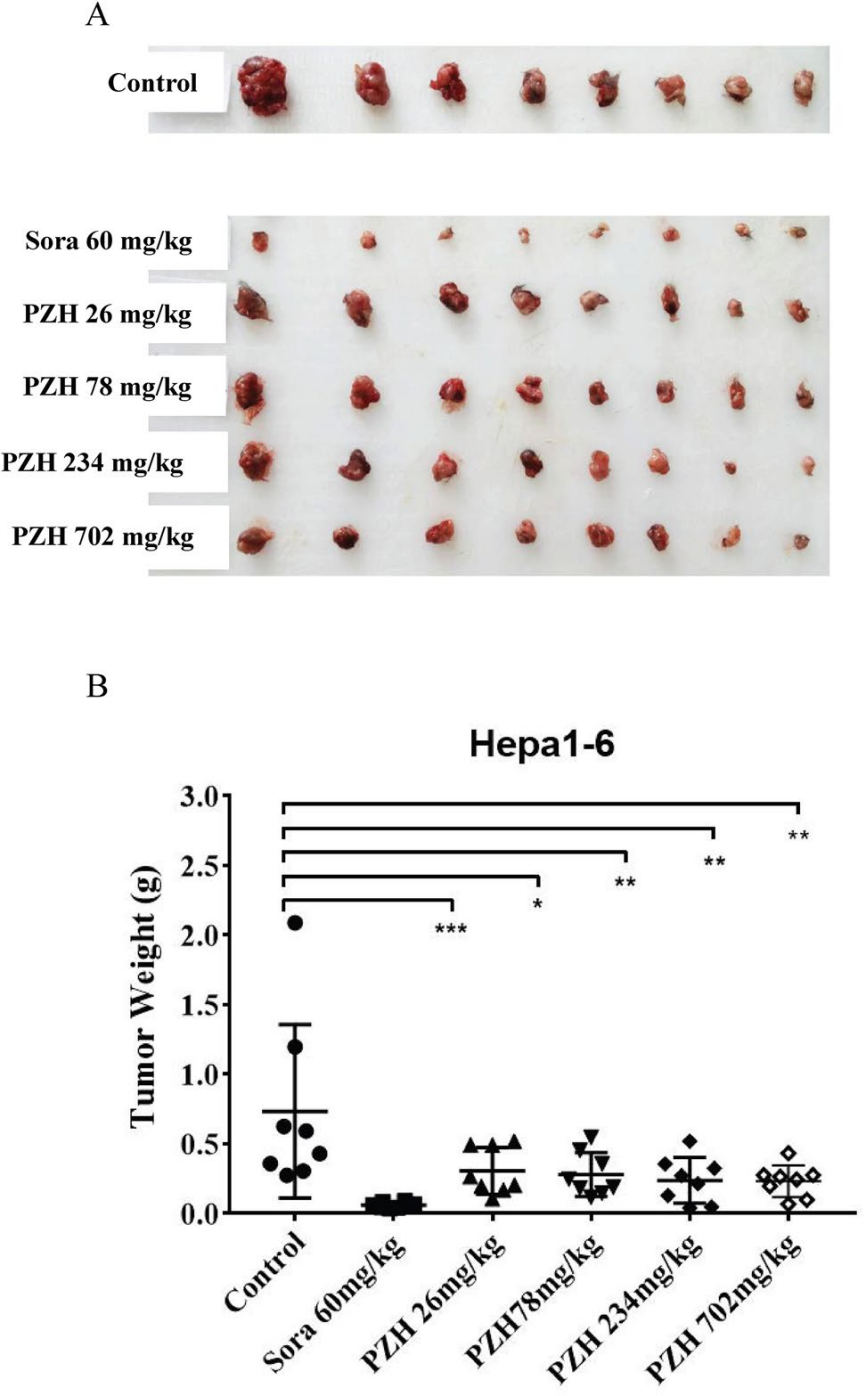
Inhibition of Pien Tze Huang (PZH) on xenograft tumor growth in vivo. C57BL/6J mice were inoculated with 5.0 × 10^6^ Hepa1-6 cells, PZH (26–702 mg/kg/day), sora (60 mg/kg/day), and 0.5% CMC as control were supplied one time per day for 21 consecutive oral administrations the next day after inoculation, then the tumor xenografts were excised completely from tissues. (**A**) A macroscopic view of the xenografted tumors after drugs’ treatments. (**B**) Analysis of tumor weight inhibition rate, n = 8, mean ± SD, one-way ANOVA, *p < 0.05 versus control group, **p < 0.01 versus control group, ***p < 0.001 versus control group.

After three weeks’ administrations of drugs, the body weight and tumor growth inhibition of each group were calculated and listed in Table 1 and Fig. 1. There was no significant difference in net weight among three groups, while the tumor weight and volume treated with sora and PZH reduced obviously compared with control group. The tumor inhibition rates of PZH at doses of 26 mg/kg, 78 mg/kg, 234 mg/kg and 702 mg/kg were 58.4%, 62.1%, 67.8% and 68.6%, respectively, which were all statistically significant. The animal dose which converted from clinical usage of PZH was 234 mg/kg, hence, this dose was selected for further study.

**Table 1:** Anti-tumor efficacy of PZH on Hepa1-6 mouse liver cancer in vivo.

Group	Dose (mg/kg × days), administration	Number (Day 1/22) n = 8	Body weight (g) Mean ± SD		Tumor weight (g)	
			Day 1	Day 22	Mean ± SD	TGI%
Control	0 × 21, po	8/8	19.6 ± 0.6	20.9 ± 0.7	0.73 ± 0.62	NA
Sorafenib	60 × 21, po	8/8	19.7 ± 0.3	19.9 ± 0.8	0.06 ± 0.02***	92.1
PZH	26 × 21, po	8/8	19.8 ± 0.4	19.5 ± 1.7	0.30 ± 0.17*	58.4
	78 × 21, po	8/8	19.9 ± 0.8	19.7 ± 1.0	0.28 ± 0.16**	62.1
	234 × 21, po	8/8	19.4 ± 0.7	19.4 ± 1.2	0.24 ± 0.16**	67.8
	702 × 21, po	8/8	18.7 ± 0.4	19.3 ± 1.3	0.23 ± 0.11**	68.6

One-way ANOVA, compared with control group, *p < 0.05, **p < 0.01, ***p < 0.001.

*NA* not applicable, *n* number of replicates, *TGI* tumor growth inhibition.

TGI % = (1 − tumor weight of treatment group/tumor weight of control group) × 100%.

For further (phospho)proteomics analysis and PRM verification, another two batches of pharmacological experiments containing control group, PZH group, and sora group were carried out, and the obtained tumor inhibition rates were 59% ± 14% and 64% ± 10% for PZH, 91% ± 2% and 82% ± 4% for sora, the body weights in control, sora, and PZH groups also did not change significantly.

### Analysis of TMT proteomics

**Figure 2:**
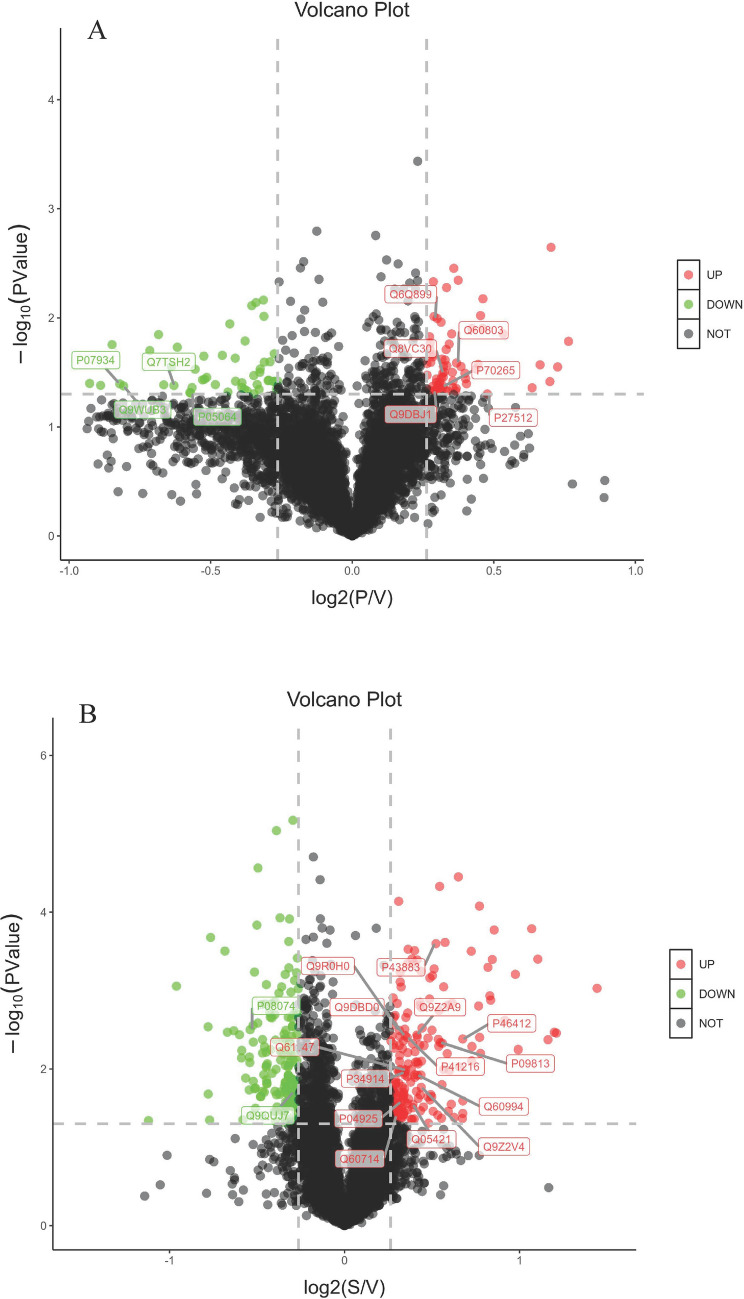
Volcano plots of proteomics treated with PZH (**A**) and sora (**B**) versus control group. In volcano plots, red and green spots represent differentially expression proteins (fold change > 1.2, *p* value < 0.05), and black spots represent the proteins changing insignificantly. The proteins of enriched KEGG pathways (fructose and mannose metabolism, glucagon signaling pathway and NF-kappa B signaling pathway in PZH vs. Control group, PPAR signaling pathway, arachidonic acid metabolism, and ferroptosis in Sora vs. Control group) were marked in the volcano maps, these proteins were selected to further PRM verification.

To gain a global view of anti-tumor effects of PZH and sora on HCC, protein expressions of the above-mentioned samples included in three independent biological replicates in each group were analyzed by using TMT-labeled proteomic study. From the proteomic results, a total of 5569 proteins in PZH, sora and solvent control groups were obtained. Among them, there were 65 up-regulated and 51 down-regulated proteins expressed differentially in the PZH versus control group according to the expression fold changing more than 1.2 or less than 0.83 times with *p* value < 0.05, and 187 up-regulated and 167 down-regulated proteins in the sora versus control group, as presented in volcano plots (Fig. 2A,B).

**Figure 3:**
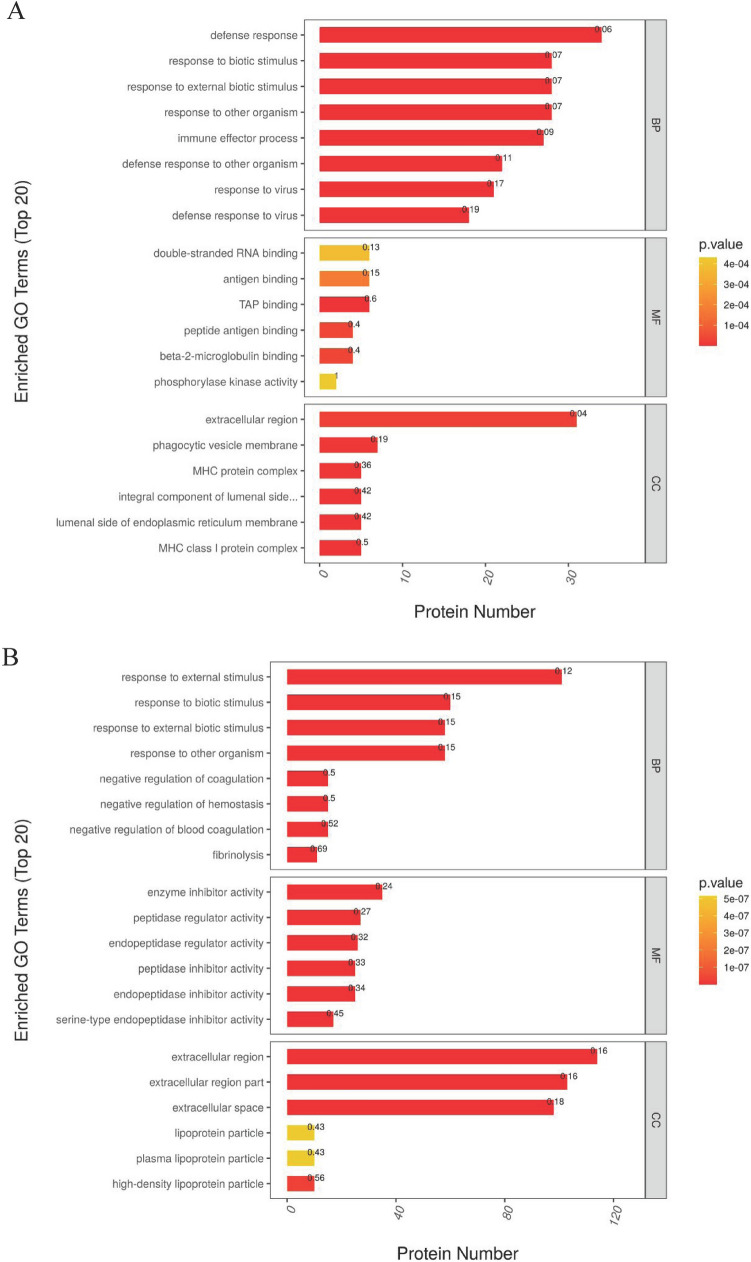
Gene ontology (GO) functional enrichment analysis of differentially expressed protein of proteomics treated with PZH (**A**) and sora (**B**). Functional classification is divided into biological process (BP), molecular function (MF) and cellular component (CC). Red represents the smaller *p* value than orange, corresponding to the higher significance level of the enrichment of the functional category. The label above the bar graph shows the enrichment factor, which indicates the ratio of differentially expressed proteins to all identified and annotated proteins.

**Figure 4:**
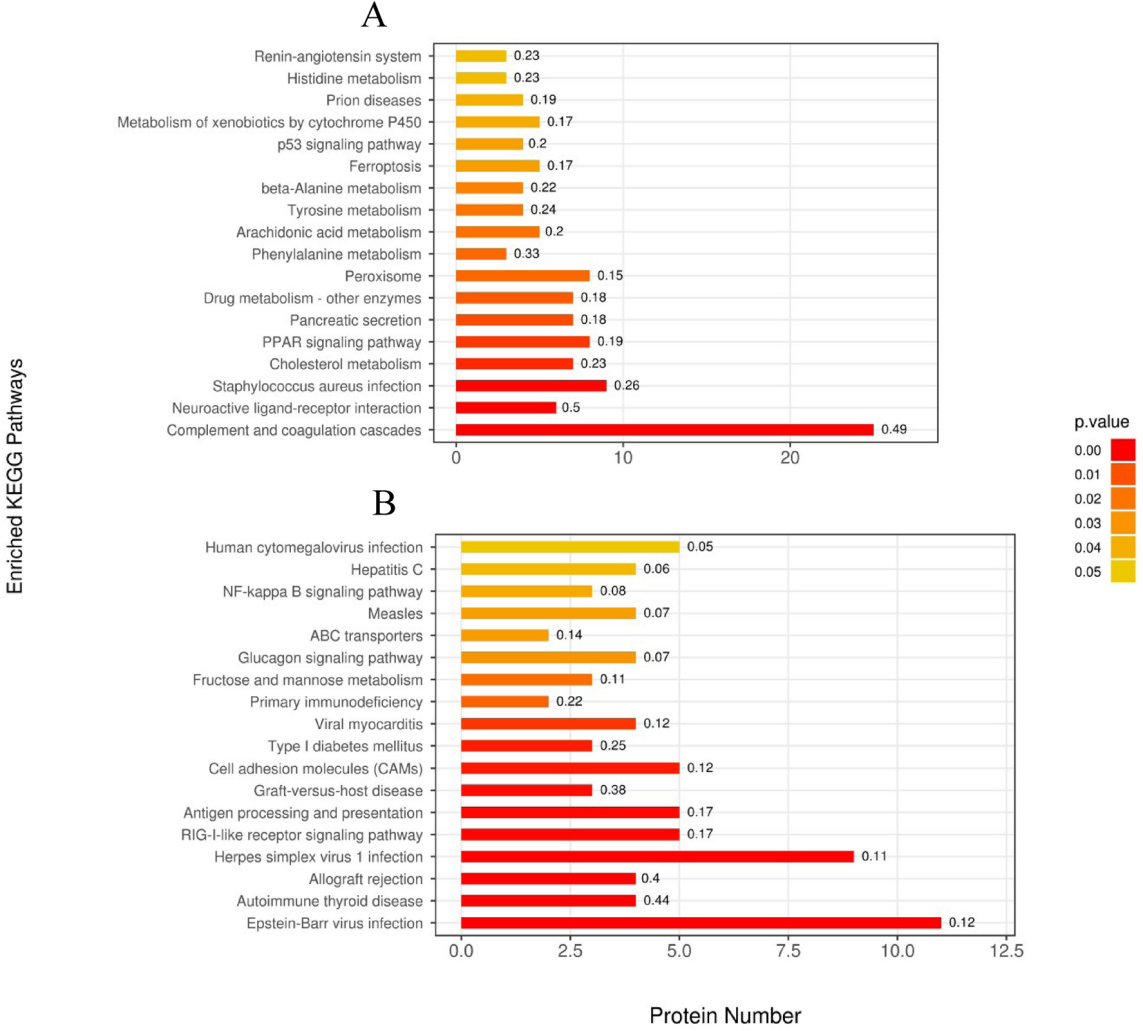
KEGG pathway enrichment analysis of proteomics treated with PZH (**A**) and sora (**B**). The ordinate represents the significantly enriched KEGG pathway, the abscissa represents the number of differentially expressed proteins contained in each KEGG pathway. The color gradient represents the size of the *p* value, the closer to red, and the smaller of *p* value, which corresponds to the enrichment degree of the KEGG pathway. The label above the bar graph shows the enrichment factor which indicates the ratio of differentially expressed proteins to the identified proteins involved in the pathway.

Through GO function annotation displayed in Fig. 3, it was found that the functions of these differential expression proteins in PZH (Fig. 3A) and sora (Fig. 3B) versus control group were mainly immune effector process, defense response, response to external biotic stimulus, response to external stimulus, and response to stimulus. Meanwhile, data from KEGG analysis indicated that pathways such as RIG-I-like receptor signaling pathway, fructose/mannose metabolism, glucagon signaling pathway, and NF-kappa B signaling pathway, were significantly enriched in response to PZH (Fig. 4A), and it was noticed that ALDOA in pathways of fructose/mannose metabolism and glycolysis/gluconeogenesis and DDX58 (probable ATP-dependent RNA helicase) in pathways of RIG-I-like receptor and NF-kappa B signaling had a high degree of protein connectivity based on protein interaction network analysis (as supplemented data of TMT proteomics). Pathways such as PPAR signaling pathway, arachidonic acid metabolism, amino acid metabolism, ferroptosis, p53 signaling pathway, were enriched in sora versus control group (Fig. 4B).

The proteomic studies suggested that anti-HCC therapies sora and PZH might participate in regulating similar biological processes, and influence protein expression of metabolic related pathways, but the regulating extent of PZH on HCC protein expression was less than sora.

### Analysis of TMT phosphoproteomics

To further explore the various roles of PZH and sora in HCC, protein phosphorylation was profiled by using quantitative TMT phosphoproteomic approach. A total of 12,783 phosphorylation sites included in 8491 unique peptides of 3576 proteins were identified. Among them, the quantities of phosphoserine (pSer), phosphothreonine (pThr) and phosphotyrosine (pTyr) were 3423 (80.77%), 683 (15.79%), and 44 (3.44%) respectively, and the average distribution number of modified sites was 3.28 per hundred amino acids with 60.37% of phosphorylated proteins contained more than two phosphorylation sites.

**Figure 5:**
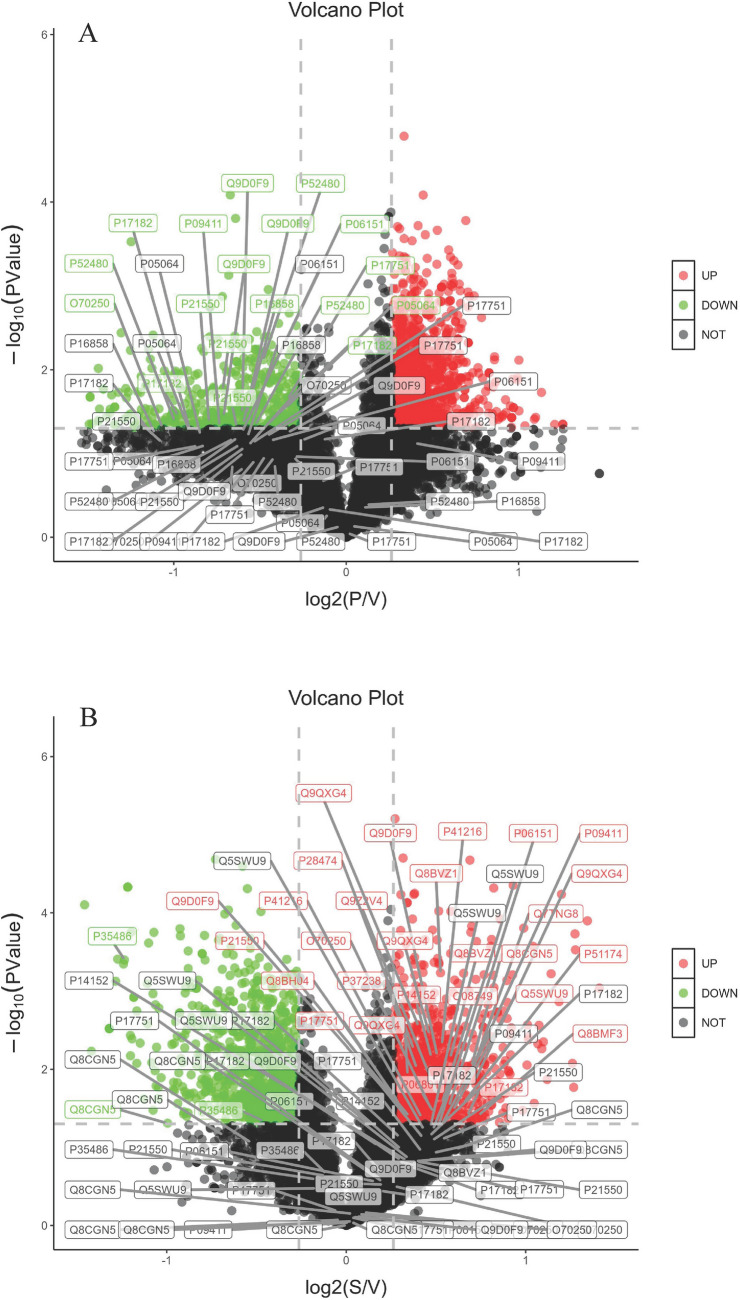
Volcano plots of phosphoproteomics treated with PZH (**A**) and sora (**B**) versus control group. In volcano plots, red and green spots represent differentially expression phosphorylated peptides (fold change > 1.2, *p* value < 0.05), and black spots represent the phosphorylated peptides changing insignificantly. The peptides whose corresponding proteins were on the enriched KEGG pathways (Glycolysis/Gluconeogenesis in PZH vs. Control group, Glycolysis/Gluconeogenesis, pyruvate metabolism, PPAR signaling pathway in Sora vs. Control group) were marked in the volcano maps, some peptides of these proteins were selected to further PRM verification.

The differentially phosphorylated peptides were calculated based on the expression folds changing more than 1.2 or less than 0.83 times with *p* value < 0.05, it was observed that 1187 phosphorylated peptides increased and 483 decreased significantly in PZH versus control group, while 867 increased and 929 decreased in sora versus control group. The quantitative results of the proteins corresponding to differentially phosphorylated peptides in three biological repetitions were presented as volcano plots (Fig. 5A,B).

**Figure 6:**
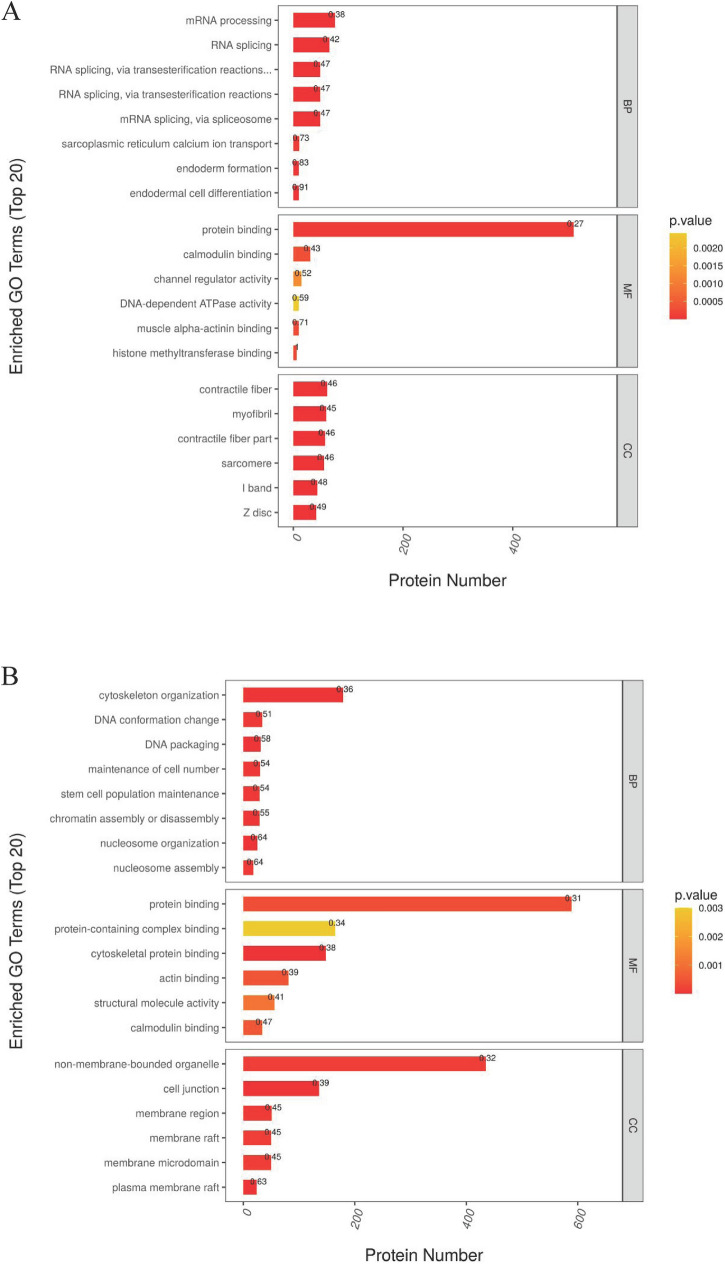
Gene Ontology (GO) functional enrichment analysis of differentially expressed protein of phosphoproteomics treated with PZH (**A**) and sora (**B**). Functional classification is divided into biological process (BP), molecular function (MF) and cellular component (CC). Red represents the smaller *p* value than orange, corresponding to the higher significance level of the enrichment of the functional category. The label above the bar graph shows the enrichment factor, which indicates the ratio of proteins of differentially expressed phosphorylated peptide to identified and annotated phosphorylated proteins.

**Figure 7:**
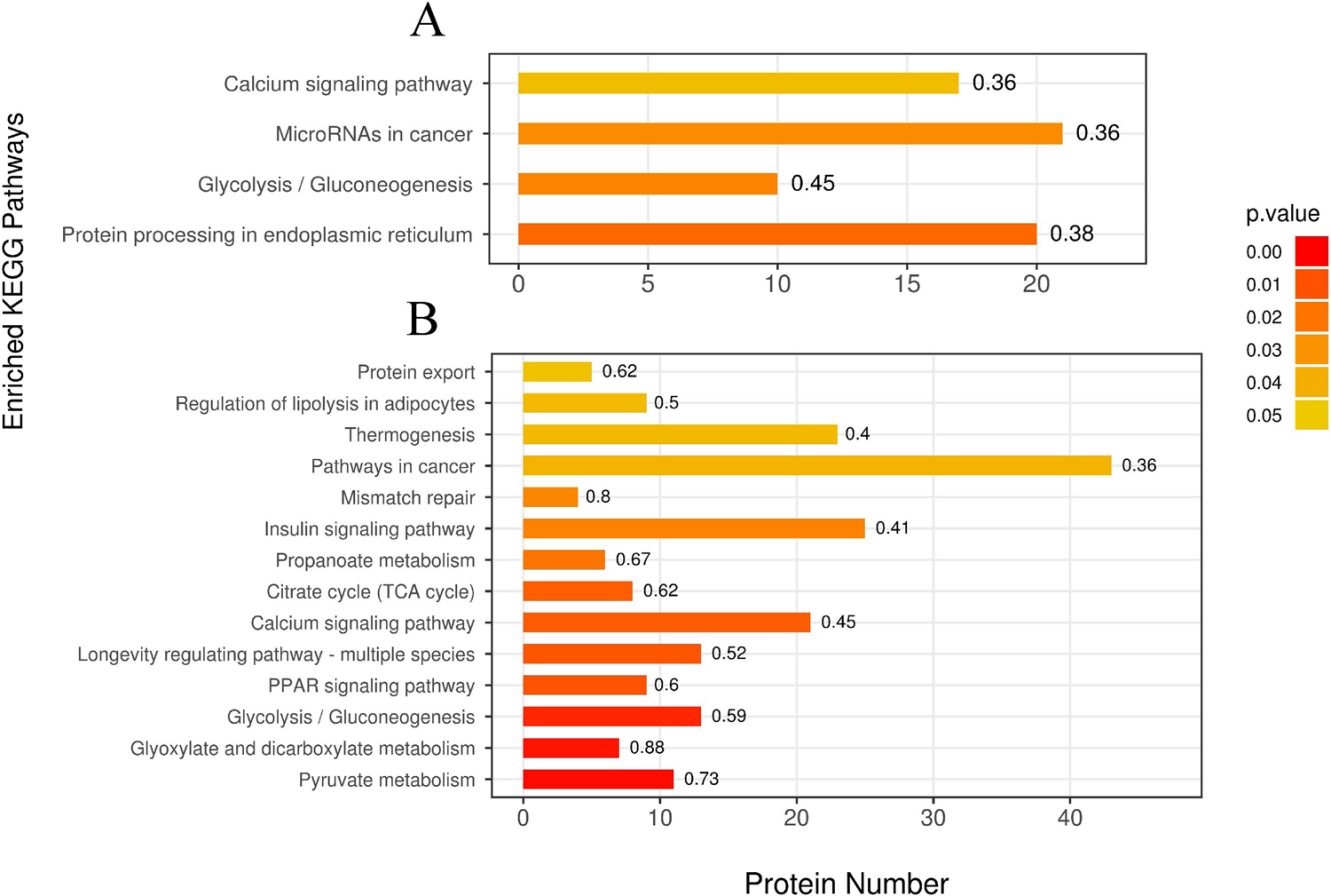
KEGG pathway enrichment analysis of phosphoproteomics treated with PZH (**A**) and sora (**B**). The ordinate represents the significantly enriched KEGG pathway, the abscissa represents the number of differentially expressed proteins contained in each KEGG pathway. The color gradient represents the size of the P value, the closer to red, the smaller of *p* value, which corresponds to the enrichment degree of the KEGG pathway. The label above the bar graph shows the enrichment factor which indicates the ratio of differentially expressed phosphorylated proteins to the identified phosphorylated proteins involved in the pathway.

Meanwhile, based on GO function results given in Fig. 6, it was found that both PZH and sora could regulate phosphorylation peptides corresponding to the molecular functions of protein binding and calmodulin binding. KEGG analysis revealed that pathways including protein processing in endoplasmic reticulum, glycolysis/gluconeogenesis, microRNAs in cancer, and calcium signaling pathway were significantly enriched in PZH versus control group (Fig. 7A), while pathways such as pyruvate metabolism, glyoxylate and dicarboxylate metabolism, glycolysis/gluconeogenesis, PPAR signaling pathway, calcium signaling pathway, citrate cycle, propanoate metabolism, and insulin signaling pathway were remarkably enriched in sora versus control group (Fig. 7B). It was noticed that ALDOA in pathways of fructose/mannose metabolism and glycolysis/gluconeogenesis, PGAM2 and LDHA in glycolysis/gluconeogenesis/glucagon signaling pathway had a high degree of protein connectivity based on protein interaction network analysis in PZH group (as supplemented data of TMT phosphoproteomics). The results again indicated that metabolic related proteins might be potential regulating sites of PZH and sora.

### PRM verification

Metabolic change plays a pivotal role in tumorigenesis and tumor progression, specifically Warburg effect represented by glycolysis has been considered as a critical trend for anti-tumor research. To ascertain whether proteins and phosphorylation sites of great interest obtained from TMT (phospho)proteomics connecting with metabolic pathways contributes to the effect of PZH and sora, a fast and effective method of targeted mass spectrometry approach namely PRM, was further used to validate the selected candidate proteins and peptides from large-scale discovery of (phospho)proteomics study.

### Confirmation of TMT proteomic results

Seven differently expression candidate proteins related to tumor metabolism based on TMT proteomic study, such as PCK1 (phosphoenolpyruvate carboxykinase) in gluconeogenesis pathway, PGAM1(phosphoglycerate mutase 1), PGAM2 (phosphoglycerate mutase 2), PDHB (pyruvate dehydrogenase E1) and LDHA (L-lactate dehydrogenase A chain) in glycolysis/gluconeogenesis, ALDOA in fructose/mannose metabolism and glycolysis/gluconeogenesis, ACSL1 (long chain fatty acid CoA ligase 1) in fatty acid biosynthesis/degradation, were chosen to verify the TMT results by liquid chromatography-PRM-mass spectrometry analysis (PRM verification). Comparisons of the above-mentioned proteins treated with PZH and sora in TMT proteomic study and PRM verification were listed in Table 2, and it was found that most selected proteins in PZH or sora versus control group verified by PRM displayed down-regulating trends, which was basically consistent with the results of TMT proteomic study, but not significant. The inconsistent significance of TMT proteomic study and PRM verification might be the parallelism of the tested samples, and indicated that expression level of these proteins might be not a key regulating target with the treatment of PZH.

**Table 2:** Comparisons of differentially expressed proteins related to metabolic pathways treated with PZH and sorafenib from TMT proteomics and PRM verification.

Uniprot	KEGG pathways	Protein description	Protein fold change					
			PZH versus Control		Sora versus Control		PZH versus Sora	
			TMT Proteomics	PRM	TMT Proteomics	PRM	TMT Proteomics	PRM
Q9Z2V4	Glucagon signaling pathway PPAR signaling pathway	Phosphoenolpyruvate carboxykinase (PCK1)	0.97	0.56	1.35*	1.62	0.71*	0.34
Q9DBJ1	Glucagon signaling pathway Glycolysis/gluconeogenesis	Phosphoglycerate mutase 1 (PGAM1)	1.23*	0.82	0.90	0.67	1.37**	1.23
O70250		Phosphoglycerate mutase 2 (PGAM2)	0.56	0.82	1.18	0.84	0.48**	0.82
Q9D051		Pyruvate dehydrogenase E1 (PDHB)	0.80	0.41	1.15	1.40	0.70	0.41*
P06151		L-lactate dehydrogenase A chain (LDHA)	0.82	0.87	1.05	0.89	0.78**	0.87
P05064	Fructose and mannose metabolism Glycolysis/gluconeogenesis	Fructose bisphosphate aldolase A (ALDOA)	0.67*	0.75	1.04	0.90	0.65**	0.75
P41216	PPAR signaling pathway Fatty acid biosynthesis/degradation	Long chain fatty acid CoA ligase 1 (ACSL1)	0.90	0.55	1.28**	1.17	0.70*	0.55

One-way ANOVA, *p < 0.05 versus control group, **p < 0.01 versus control group. The value greater than 1 indicates up-regulation, less than 1 indicates down-regulation.

### Confirmation of TMT phosphoproteomic results

**Figure 8:**
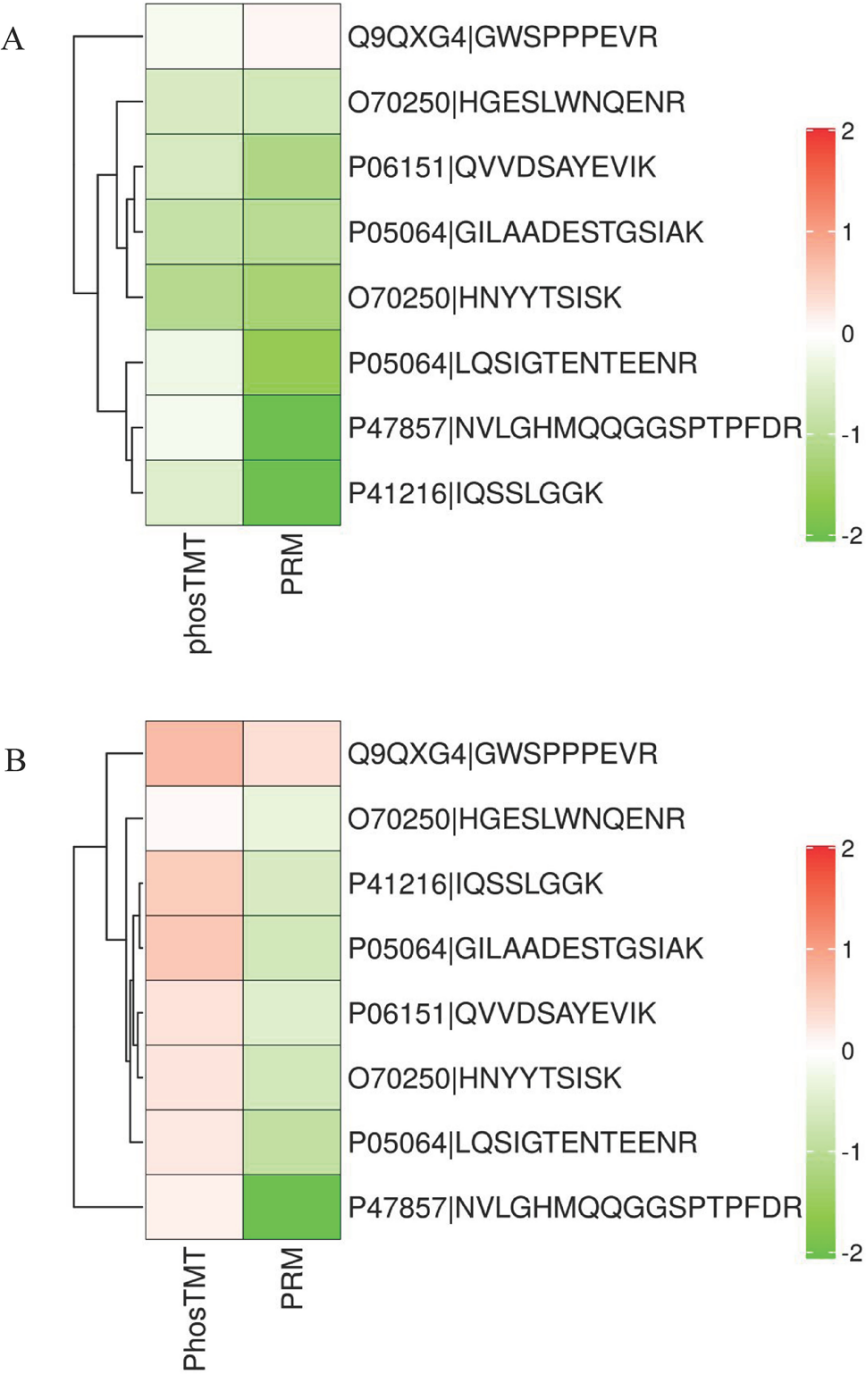
Heat map summary of 8 selected phosphopeptides in PRM verification (right) and TMT phosphoproteomics (left) treated with PZH (**A**) and sorafinib (**B**). In the heatmaps, average expression level of 8 phosphorylated peptides displayed in Table 3 were presented as different colour, where red represents significantly up-regulated phosphorylated peptide, green represents significantly down-regulated phosphorylated peptide.

Concluded from the TMT phosphoproteomic results, differentially expressed phosphorylation sites in peptides of PGAM2 and LDHA (in glycolysis/gluconeogenesis), PFK1 and ALDOA (in glycolysis/gluconeogenesis, and fructose/mannose metabolism), ACSS2 (acetyl-coenzyme A synthetase in pyruvate metabolism, propanoate metabolism and glycolysis/gluconeogenesis), and ACSL1 (in fatty acid biosynthesis/degradation), were selected for PRM verification. Basically, all selected phosphorylation sites except ACSS2 displayed obvious down-regulated in PZH versus control and sora versus control, as listed in Table 3. Therein, the changing trends of seven phosphorylation sites of five proteins verified by PRM were consistent with the TMT phosphoproteomic anylysis in PZH versus control, while the changing trends of six phosphorylation sites of five proteins were contrary to the TMT phosphoproteomic results in sora versus control. The comparions of PRM verification and TMT phosphoproteomics of the selected peptides treated with PZH and sorafinib were summarized in heatmaps, as depicted in Fig. 8. Albeit the PRM results showed that both PZH and sora could inhibit the protein phosphorylation of glycolytic pathway, this opposite verification trends different between PZH and sora group implied that the regulating role of these proteins in glycolysis pathway might be more continuous and stable or significant to PZH treatment compared to sora.

**Table 3:** Comparisons of differentially expressed phosphorylated peptides related to metabolic pathways treated with PZH and sorafenib from TMT phosphoproteomics and PRM verification.

Uniprot	KEGG pathways	Protein description Peptide name	Phosphorylation fold change					
			PZH versus control		Sora versus control		PZH versus sora	
			TMT Phospho proteomics	PRM	TMT Phospho proteomics	PRM	TMT Phospho proteomics	PRM
O70250	Glucagon signaling pathway Glycolysis/gluconeogenesis	Phosphoglycerate mutase 2 (PGAM2)	\	\	\	\	\	\
		*Peptide (residues 129–139)*: HGE**S**LWNQENR	0.68#	0.63**	0.88*	0.79*	0.64**	0.80#
		*Peptide (residues 12–20)*: HN**Y**YTSISK	0.48*	0.41**	1.21	0.64**	0.55**	0.63*
P06151		L-lactate dehydrogenase A chain (LDHA)	\	\	\	\	\	\
		*Peptide (residues 233–243)*: QVVDSA**Y**EVIK	0.66	0.45**	1.23	0.72*	0.53**	0.62**
P47857	Glycolysis/gluconeogenesis HIF-1 signaling pathway Fructose and mannose metabolism	ATP dependent 6-phosphofructokinase (PFK1)	\	\	\	\	\	\
		*Peptide (residues 657–673)*: NVLGHMQQGG**S**PTPFDR	0.89	0.25**	1.12	0.23**	0.79*	1.09
P05064		Fructose bisphosphate aldolase A (ALDOA)	\	\	\	\	\	\
		*Peptide (residues 44–56)*: LQ**S**IGTENTEENR	0.84	0.34**	1.18*	0.54*	0.71**	0.53*
		*Peptide (residues 29–42)*: GILAADE**S**TGSIAK	0.57	0.49**	1.50*	0.64**	0.45**	0.76*
Q9QXG4	Glycolysis/gluconeogenesis Pyruvate, propanoate metabolism	Acetyl-coenzyme A synthetase (ACSS2)	\	\	\	\	\	\
		*Peptide (residues 28–36)*: GW**S**PPPEVR	0.89	1.08	1.32**	1.26*	0.54*	0.86
P41216	PPAR signaling pathway Fatty acid biosynthesis/degradation	Long chain fatty acid CoA ligase (ACSL1)	\	\	\	\	\	\
		*Peptide (residues 421–428)*: IQS**S**LGGK	0.71#	0.25**	1.34*	0.68	0.53**	0.37**

One-way ANOVA, *p < 0.05 versus control group, **p < 0.01 versus control group. The value greater than 1 indicates up-regulation, less than 1 indicates down-regulation.

## Discussion

Metabolic reprogramming is common in solid tumors and emerged as an essential contributor to the tumor progression machinery, especially aerobic glycolysis which known as the Warburg effect. A better understanding of glycolysis with anti-tumor drug will serve to understand the pathogenesis and curative mechanism, as well as to improve the deficiencies of medication^7^. TCM could inhibit cancer growth and metabolism by decreasing the expression level of glycolytic enzymes LDH, HKII and GLUT1, ENO1 in various cancers^22,23^. However, detailed phosphorylations of enzymes in glycolytic pathway treated with TCM were seldom reported^25,26^, albeit delineation of tissue phosphoproteome is pivotal to unravel the underlying mechanisms of diseases with deregulated cellular signaling.

PZH is a famous TCM, which was reported to inhibit the growth of HCC cells^27^ and bring significant clinical efficacy^17–19^. In the present study, we confirmed that PZH and sora could inhibit the HCC tumor growth in Hepa1-6 cell implanted mice model. Based on subsequently analysis of (phospho)proteomics and PRM verification, as summarized in Table 3 and Fig. 8, it was convinced that peptide phosphorylation instead of protein expression of PFK1, ALDOA, PGAM2, and LDHA in glycolytic pathway were substantially repressed for about 2–4 folds by PZH and sora, and phosphorylation of ACSL1 related to fatty acid biosynthesis/degradation pathway was inhibited for fourfold by PZH but not sora, while phosphorylation of ACSS2 was up-regulated for 1.3-fold by sora which changed insignificantly in PZH group.

PFK1, the first committing step of glycolysis, catalyzes the phosphorylation of D-fructose 6-phosphate to fructose 1,6-bisphosphate by ATP. Its activity has been found to promote glycolysis and proliferation in cancer cells and was correlated with poor prognosis or survival^7^. S667 phosphorylation of PFK1 has been annotated in UniProt website (https://www.uniprot.org/), and it was reported that phosphorylation of PFKP (another isoform of PFK1) Y64 could enhance PFK1 activation and GLUT1 expression which promoted the Warburg effect, tumor cell proliferation, and brain tumorigenesis^28^, and phosphorylation of PFK1 platelet isoform PFKP S386 in human glioblastoma cells could increase PFKP expression and promote aerobic glycolysis, cell proliferation, and brain tumor growth^29^. In this study, phosphorylation of PFKM (one of the PFK1 isoforms) decreased for fourfold and 4.3-fold with the treatment of PZH and sora respectively, but it remains elusive of S667 phosphorylation function or if PFKM activity was down-regulated by PZH and sora through serine phosphorylation to attenuate Warburg effect and tumor growth.

ALDOA, the most abundant aldolase isoform in cancers, plays a key role in glycolysis and gluconeogenesis. Phosphorylations of S36 and S46 in ALDOA have been annotated in the website of UniProt which inferred from the combination of experimental and computational evidence. In this study, we verified that phosphorylations of these two sites reduced for about 2.9-fold and 2.0-fold respectively under the condition of PZH, while they decreased for 1.9-fold and 1.6-fold in sora group. The down-regulated phosphorylated levels of ALDOA peptides in HCC proposed that impairment of glycolytic phenotype might be a distinguished feature of HCC treated with PZH or sora. Previous studies have reported that ALDOA was a critical driver for HCC cell growth under hypoxia, and considered as a promising therapeutic strategy for treating HCC^30,31^, and a study using paired tumor and adjacent liver tissues of hepatitis B virus-related HCC patients revealed that phosphorylation of glycolytic enzymes including ALDOA might drive metabolic reprogramming and proliferation in beta-catenin-mutated HCC^32^. However, whether PZH directly affects enzyme activity or through regulating the upstream signaling pathway of ALDOA remains to be clarified.

PGAM catalyzes the conversion of 3-phosphoglycerate into 2-phosphoglycerate in the late stage of glycolysis. PGAM1 and PGAM2 are two tissue-specific isoforms in human, the former has been reported to be commonly upregulated in many human cancers, and was induced to play an important role in mTOR-mediated Warburg effect and tumor growth, whose abundance was an adverse predictor for patient survival in NSCLC, while the latter had no above-mentioned functions^33^ and its relationship with tumors was seldom reported. Our study verified that two phosphorylation sites (S14, Y132) of PGAM2 were significantly down-regulated for 1.6-fold and 2.4-fold with the treatment of PZH, for 1.3-fold and 1.6-fold by sora, this result was consistent with the reported phosphorylated sites of PGAM2 which did not show the potential impact of serine phosphorylation on enzyme activity^34^. It was reported that an *Arabidopsis thaliana* 2,3-bisphosphoglycerate-independent PGAM (iPGAM) phosphopeptide contained a phosphorylated serine 80 and serine 82 in iPGAM1 and iPGAM2 respectively, and it was verified that the metabolic consequences of S82 phosphorylation was a higher iPGAM enzyme activity in light-treated compared to light/dark-treated *A. thaliana* rosette leaves^35^. Meanwhile, it was demonstrated that the phosphorylation of tyrosine 26 residue of PGAM1 greatly enhances its activity by enhancing the binding of PGAM1 to its substrates, and thus upregulates glycolysis and promotes tumor growth^36^. However, the exact effect of PZH on PGAM2 enzyme in this study is far unknown based on the seldom publication.

LDHA, an isoenzyme of lactate dehydrogenase, functions in the final step of glycolysis to convert pyruvate to lactate, and was reported to be up-regulated in tumors and as a marker of prognosis of cancer patients^37^. Changes of oligomeric state, stability and catalytic activity were reported due to post-translational modifications of LDHA^38^, therein, phosphorylation of LDHA Y239 was the first report to associate with chromatin which promotes a damage response transcriptional program. Tyrosine phosphorylation of LDHA increased its enzymatic activity by enhancing tetramerization and increasing cofactor association to promote the Warburg effect and tumor growth^38,39^. In this study, the similar phenomenon was also found, phosphorylation of Y239 in LDHA was observed to diminish for 2.2-fold and 1.4-fold with the phenotype of tumor inhibition in PZH and sora groups compared to control sample. It was reported that tyrosine residues of LDHA were subject to phosphorylation by various kinases in cancer cells and provided metabolic advantages to tumor growth^39,40^, but we did not notice significant changes of these signal pathways in the (phospho)proteomic study. Detailed study will be continued to explore if PZH directly inhibiting LDHA or through regulating kinase signal pathways.

Acyl-CoA synthetase long-chain family catalyzes the first step in lipid metabolism after fatty acid entry into the cell, it comprises five isoenzymes which were deregulated in various tumors and considered as a potential anti-tumor therapy^41^. ACSL1 has an obvious preference for oleate and linoleate^42^, enhancement of ACSL1 expression level was observed in breast cancer and HCC^41,43^, co-inhibitions of ACSL1 and glycolysis resulted in less cell proliferation and migration in colorectal cancer^41,44^. In this study, with the treatment of PZH, protein expression of ACSL1 was decreased but not significant, while the phosphorylation of S424 was remarkably declined for fourfold according to PRM verification, in contrast, protein abundance and S424 phosphorylation of ACSL1 were affected insignificantly in sora group based on PRM results. It was reported that S424 in endogenous ACSL1 from mouse liver and brown adipose tissue was phosphorylated^45^, but no information was revealed to connect the phosphorylation with ACSL1 activity. We inferred that PZH might exert synergistic effects of glycolysis and fatty acid activation to weaken Warburg effect, which might be a different mechanism of PZH and sora to HCC.

To further investigate the role of PZH in metabolic related pathways, multiple reaction monitoring mass spectrometry was adopted to detect the important metabolites in the tricarboxylic acid cycle, glycolytic pathway, and oxidative phosphorylation process in HCC tumor tissues. It was observed that the accumulations of key metabolites in the above mentioned pathways showed no significant change in PZH group compared to control (as supplementary data of energy metabolites detection). However, by using selected ion monitor method to detect C6-C24 fatty acids, the levels of arachidonate and docosatetraenoate instead of oleate and linoleate (the reported substrates of ACSL1) were found to enhance for 1.2-fold and 1.3-fold respectively in response to PZH treatment (as supplementary data of long and medium fatty acids detection), this phenomenon was also reported in other drug-treated tumors which displayed an increase in the level of polyunsaturated fatty acids^46,47^. We inferred that arachidonate and/or docosatetraenoate might be another preferential substrates of ACSL1, and their accumulations might be due to the down-regulating phosphorylation of ACSL1.

In summary, the present study is the first time to compare the potential anti-tumor mechanisms of PZH and sorafenib in regulating the phosphorylation of enzymes related to cancer metabolic pathway by combining TMT and PRM mass spectrometry, and it was found that both PZH and sora could participate in regulating the phosphorylation of enzymes PGAM2, PFK1, ALDOA, and LDHA in metabolic related pathways, while sora and PZH have no effect on the phosphorylation of ACSL1 and ACSS2, respectively.

We speculated that the traditional Chinese medicine PZH might target phosphorylation of enzymes in metabolic related pathways instead of protein abundance to inhibit HCC, which might share a similar mechanism of anti-HCC agent of sorafenib in regulating glycolytic pathway, but differing in regulating fatty acid biosynthesis/degradation pathway. This finding supplies an underlying molecular basis for PZH acting as a TCM therapy or combination with chemotherapy drugs for tumors. Further systematic study focusing on more metabolic related genes/enzymes and metabolites will be continued to further uncover the precise anti-tumor mechanism of PZH treatment.

## Materials and methods

### Drug, cell, and animal

PZH (batch number 1607039) was produced and authenticated by Zhangzhou Pien Tze Huang Pharmaceutical Co., Ltd. (China Food and Drug Administration approval No. Z35020243, Zhangzhou, China). Positive control drugs sorafenib tosylate tablet (batch number BXHTUE2, hereafter referred to as sora) was purchased from Byer Pharma AG.

Mouse hepatocarcinoma cell line Hepa1-6, purchased from the Institute of Basic Medicine, Chinese Academy of Medical Sciences, was cultured and passaged in 10% FBS DMEM medium.

C57BL/6J mice (SPF grade, female), weighing 18–20 g, were provided by Sibefu (Beijing) Biotechnology Co., Ltd.. The laboratory animal quality certificate number is 1103241911028976, the laboratory animal production license number is SCXK (Jing) 2019-0010, the laboratory animal license number is SYXK (Beijing) 2014-0023, and the experimental condition was shielded environment. All procedures were approved by the Ethics Committee for Animal Experiments of the Institute of Materia Medica, Chinese Academy of Medical Sciences & Peking Union Medical College and conducted under the Guidelines for Animal Experiments of Peking Union Medical College.

### Implantation of tumors and treatment

Hepa1-6 cells were collected under aseptic condition, and diluted with normal saline to obtain the tumor cell suspension with a concentration of 1.0 × 10^7^ cells/mL. Suspension was then inoculated in the right armpit of C57BL/6 mice (2.0 × 10^6^ cells, 0.2 mL/mouse), and recorded as day 0. The animals were randomly divided into six groups (eight mice each group) the next day after inoculation, weighed and administered. The solvent control group was given 0.5% CMC orally every day, and the test drug PZH (concentrations of 26, 78, 234 and 702 mg/kg/day) and positive control compound sora (concentration of 60 mg/kg/day) were supplied one time per day for 21 consecutive oral administrations (0.4 mL each mouse). At the termination of experiments, all animals were sacrificed and weighted, and the tumor tissues were stripped, weighed and photographed. The tumor inhibition rate was calculated according to the following formula to evaluate the intensity of the anti-tumor effect: TGI % = (1 − tumor weight of treatment group/tumor weight of control group) × 100%.

For further studies of (phospho)proteomics analysis and PRM verification, samples were prepared from another two batches of implanted mice, each batch was randomly divided into three groups (twelve mice in each group) as the solvent control group, the test drug PZH group (234 mg/kg) and the positive drug sora group (60 mg/kg).

### TMT (Phospho)proteomics analysis and PRM verification

The tumor tissue samples of each group were randomly selected and mixed into three biological repeats (four samples mixed into one repeat) to apply for protein extraction, TMT (phospho)proteomics analysis and PRM verification were conducted as follow.

### Sample preparation and LC–MS/MS separation of TMT (Phospho)proteomics analysis

(Phospho)proteomics experiments mainly included protein extraction, peptide enzymolysis, TMT labeling, phosphorylated peptide enrichment (only in phosphoproteomics), liquid chromatography-tandem mass spectrometry (LC–MS/MS) data collection, and proteins or phosphorylated peptide identification and quantitative analysis, screening of differentially expressed proteins or proteins corresponding to differentially expressed phosphorylated peptides, function annotation and pathway analysis, and clustering analysis of differentially expressed phosphorylated peptides and proteins.

Sample preparation briefly as following: the protein of the tumor tissues was first lysed by SDT buffer (4% (w/v) sodium dodecyl sulphate, 100 mM Tris/HCl pH7.6, 1 mM dithiothreitol), and quantified by BCA method. Appropriate amount of protein from each sample was then trypsinized with FASP (filter aided sample preparation) method, and peptides were quantified by detecting OD_280_. For TMT phosphoproteomic analysis, beofore quantified by detecting OD_280_, C18 Cartridge was used to desalt the enzymatically hydrolyzed peptide segment, and 40 μl dissolution buffer was added after freeze-drying. After that, 100 μg peptides of each sample were labeled according to the instruction of Thermo Company's TMT labeling kit, and the labeled samples were fractionated using High pH Reversed-Phase Peptide Fractionation Kit (Thermo scientific) for proteomic analysis, or enriched by iMAC enriched method (High-Select™ Fe-NTA Phosphopeptides Enrichment Kit, Thermo Scientific) for phosphoproteomic detection.

LC–MS/MS separation briefly as follows: Each sample was separated by HPLC liquid phase system Easy nLC with nanoliter flow rate. Buffer solution A is 0.1% formic acid aqueous solution, buffer solution B is 0.1% formic acid acetonitrile (84% acetonitrile). The chromatographic column was balanced with 95% buffer A, and the sample was loaded by the automatic sampler to the loading column (Thermo Scientific Acclaim PepMap100, 100 μM * 2 cm, nanoViper C18), and separated by the analysis column (Thermo scientific EASY column, 10 cm, ID75 μm, 3 μm, C18-A2), the flow rate was 300 nL/min. After chromatographic separation, the samples were analyzed by mass spectrometry with Q-Exactive mass spectrometer. The detection method was positive ion, and the scanning range of parent ion is 300–1800 m/z, resolution of primary mass spectrum was 70,000 at 200 m/z, AGC (Automatic gain control) target was 1e6, Maximum, IT was 50 ms, and dynamic exclusion time was 60.0 s. The mass charge ratio of polypeptide and polypeptide fragments was as following: 20 MS2 scans were collected after each full scan, and the MS2 activation type was HCD, Isolation Window was 2 m/z. Resolution of secondary mass spectrometry was 17,500 at 200 m/z, Normalized Collision Energy is 30 eV, Underfill 0.1%.

The original data obtained by mass spectrometry analysis was a RAW file, further library identification and quantitative analysis were carried out on the software Mascot 2.2 and Proteome Discoverer 1.4.

### Bioinformatics analysis of TMT (Phospho)proteomics analysis

Hierarchical clustering heat maps were classified and generated by using Complex heatmap R package (R Version 3.4) from the two dimensions of sample and protein expression. Annotations of GO and KEGG pathway of the target protein collection were carried out by Blast2GO and KAAS^48^ (KEGG Automatic Annotation Server) software, and subsequently enrichment analysis was performed after comparing the distribution of GO classification or KEGG pathway. The protein–protein interaction (PPI) was retrieved in STRING software (http://string-db.org/) and analyzed by Cytoscape software (http://www.cytoscape.org/, version 3.2.1), the degree of each protein was calculated to evaluate the importance of the protein in the PPI network.

### Sample preparation and LC-PRM/MS separation of PRM verification

For PRM verification of TMT proteomic and phosphoprotemic results, the expression levels of selected proteins/peptides were further quantified by LC-PRM/MS analysis to verify the protein expression/peptide phosphorylation levels obtained by TMT label analysis.

For sample preparation, briefly, peptides were prepared according to the obove-mentioned TMT protocol, for phosphoproteomic PRM sample, and an Peptide Retention Time Calibration Mixture (Thermo Scientific) stable isotope peptides was spiked in each sample as internal standard reference.

LC-PRM/MS separation briefly as following: Tryptic peptides were loaded on C18 stage tips for desalting prior to reversed-phase chromatography on an Easy nLC-1200 system (Thermo Scientific). One hour liquid chromatography gradients with acetonitrile ranging from 5 to 35% in 45 min were used. PRM analysis was performed on a Q Exactive HF mass spectrometer (Thermo Scientific). Methods optimized for collision energy, charge state, and retention times for the most significantly regulated peptides were generated experimentally using unique peptides of high intensity and confidence for each target protein. The mass spectrometer was operated in positive ion mode and with the following parameters: The full MS1 scan was acquired with the resolution of 60,000 (at 200 m/z), automatic gain control (ACG) target values 3.0 × 10^−6^, and a 200 ms maximum ion injection times. Full MS scans were followed by 20 PRM scans at 35,000 resolution (at m/z 200) with AGC 3.0 × 10^−6^ and maximum injection time 120 ms. The targeted peptides were isolated with a 1.6 Th window. Ion activation/dissociation was performed at normalized collision energy of 27 in a higher energy dissociation (HCD) collision cell. The raw data were analyzed using Skyline 3.5.0 where signal intensities for individual peptide sequences for each of the significantly altered proteins were quantified relative to each sample and normalized to standard reference.

### Statistical analysis

All groups contained three biological samples. All statistical analyses were performed using GraphPad Prism 5, comparisons were analyzed using an independent one-way ANOVA, *p* value < 0.05 was considered statistically significant.

### Ethics approval

The animal study was approved by the Ethics Committee for Animal Experiments of the Institute of Materia Medica, Chinese Academy of Medical Sciences & Peking Union Medical College and conducted under the Guidelines for Animal Experiments of Peking Union Medical College (EC approved number was 00000529). We confirm that the study is reported in accordance with ARRIVE guidelines.

## Data Availability

The mass spectrometry data have been deposited to the ProteomeXchange Consortium (http://proteomecentral.proteomexchange.org) via the iProX partner repository with the dataset identifier PXD037540.

## Abbreviations

ANOVA: Analysis of variance
ALDOA: Fructose-bisphosphate Aldolase A
ACSL1: Long chain fatty acid CoA ligase
ACSS2: Acetyl-coenzyme A synthetase
BCA: Bicinchoninic acid
ENO1: Enolase 1
FASP: Filter aided sample preparation
GLUT1: Glucose transporter type 1
HCC: Hepatocellular carcinoma
HKII: Hexokinase 2
iMAC: Immobilized metal affinity chromatography
KEGG: Kyoto Encyclopedia of Genes and Genomes
LC–MS: Liquid chromatography–mass spectrometry
LDHA: Lactate dehydrogenase A chain
NF-kappa B: Nuclear factor kappa-B
PCK1: Phosphoenolpyruvate carboxykinase 1
PDHB: Pyruvate dehydrogenase E1
PFK1: Phosphofructokinases 1
PPAR: Peroxisome proliferator-activated receptor
PGAM2: Phosphoglycerate mutase 2
PPI: Protein–protein interaction
PRM: Parallel reaction monitoring
PZH: Pien Tze Huang
RIG-I: Retinoic acid-inducible gene-I
Sora: Sorafenib
TCM: Traditional Chinese medicine
TMT: Tandem mass tag
